# 

**Published:** 1891-10

**Authors:** 


### CONTENTS.

ORIGINAL COMMUNICATIONS—
Fracture of the Cartilaginous Portion of the Nasal Septum, Followed by Acute Traumatic Perichondritis. By Charles Dunbar Roy, A. B., M. D., Atlanta, Ga. .... 449
Sources of Animal Heat. By A. D. Barr, M. D., Calamine, Ark. .... 455
Ovariotomy during Pregnancy. By Christian Fenger, M. D., Chicago, Ill. .... 458
Two Cases of Fibroid Tumor of the Uterus Successfully Treated by Weak Currents of Galvanism. By J. A. Lyons, M. D. Chicago, Ill. .... 468
One Hundred Consecutive Cases of Skin Disease. IX. By M. B. Hutchins, M. D., Atlanta, Ga. .... 471
SOCIETY REPORTS—
Gynecological and Obstetrical Society of Baltimore .... 476
Transactions of the Gynecological Society of Chicago .... 481
CORRESPONDENCE—
Our New York Letter .... 488
IN MEMORIAM, Henry Gaither, M. D .... 492
EDITORIAL—
To Our Readers .... 498
Medical Words .... 498
SELECTIONS .... 499–509
BOOK REVIEWS—
Transactions of the Southern Gynecological Association .... 510
Standard English Dictionary .... 510

